# *N*′-(5-Chloro-2-hydroxy­benzyl­idene)nicotinohydrazide

**DOI:** 10.1107/S1600536809020819

**Published:** 2009-06-06

**Authors:** Chong-Gui Ren

**Affiliations:** a Department of Chemistry and Chemical Engineering, Zaozhuang University, Zaozhuang Shandong 277160, People’s Republic of China

## Abstract

There are two independent Schiff base mol­ecules in the asymmetric unit of the title compound, C_13_H_10_ClN_3_O_2_. The dihedral angles between the benzene and pyridine rings are 12.8 (2) and 1.9 (2)° in the two mol­ecules. Intra­molecular O—H⋯N hydrogen bonds are observed. Mol­ecules are linked into centrosymmetric *R* ^4^_4_(26) motifs by N—H⋯O and N—H⋯N inter­actions.

## Related literature

For the biological properties of Schiff base compounds, see: Jeewoth *et al.* (1999 ▶); Ren *et al.* (2002 ▶); Eltayeb *et al.* (2008 ▶); Sinha *et al.* (2008 ▶). For metal complexes of Schiff base compounds, see: Shivakumar *et al.* (2008 ▶); Prabhakaran *et al.* (2006 ▶); Dhar *et al.* (2005 ▶). For related structures, see: Cui *et al.* (2007 ▶); Jing *et al.* (2007 ▶); Ma *et al.* (2008 ▶); Salhin *et al.* (2007 ▶); Lin *et al.* (2007 ▶); Alhadi *et al.* (2008 ▶); Xue *et al.* (2008 ▶); Wang *et al.* (2008 ▶); Lu (2008 ▶); Diao *et al.* (2008 ▶); Qiu (2009 ▶); Mohd Lair *et al.* (2009*a* ▶,*b* ▶). For reference structural data, see: Allen *et al.* (1987 ▶). For hydrogen-bond motifs, see: Bernstein *et al.* (1995 ▶).
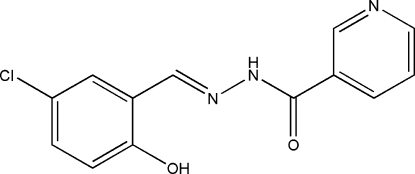


## Experimental

### 

#### Crystal data

C_13_H_10_ClN_3_O_2_
*M* *_r_* = 275.69
Monoclinic, 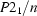

*a* = 9.792 (2) Å
*b* = 23.358 (3) Å
*c* = 10.926 (2) Å
β = 96.848 (2)°
*V* = 2481.2 (8) Å^3^
*Z* = 8
Mo *K*α radiation
μ = 0.31 mm^−1^
*T* = 298 K
0.30 × 0.30 × 0.27 mm

#### Data collection

Bruker SMART CCD area-detector diffractometer
Absorption correction: multi-scan (*SADABS*; Sheldrick, 1996 ▶) *T*_min_ = 0.913, *T*_max_ = 0.921
14387 measured reflections
5342 independent reflections
3193 reflections with *I* > 2σ(*I*)
*R*_int_ = 0.042

#### Refinement

*R*[*F* ^2^ > 2σ(*F* ^2^)] = 0.049
*wR*(*F* ^2^) = 0.115
*S* = 1.01
5342 reflections
351 parameters
2 restraints
H atoms treated by a mixture of independent and constrained refinement
Δρ_max_ = 0.22 e Å^−3^
Δρ_min_ = −0.24 e Å^−3^

### 

Data collection: *SMART* (Bruker, 2002 ▶); cell refinement: *SAINT* (Bruker, 2002 ▶); data reduction: *SAINT*; program(s) used to solve structure: *SHELXS97* (Sheldrick, 2008 ▶); program(s) used to refine structure: *SHELXL97* (Sheldrick, 2008 ▶); molecular graphics: *SHELXTL* (Sheldrick, 2008 ▶); software used to prepare material for publication: *SHELXL97*.

## Supplementary Material

Crystal structure: contains datablocks global, I. DOI: 10.1107/S1600536809020819/bx2215sup1.cif

Structure factors: contains datablocks I. DOI: 10.1107/S1600536809020819/bx2215Isup2.hkl

Additional supplementary materials:  crystallographic information; 3D view; checkCIF report

## Figures and Tables

**Table 1:** Hydrogen-bond geometry (Å, °)

*D*—H⋯*A*	*D*—H	H⋯*A*	*D*⋯*A*	*D*—H⋯*A*
O1—H1⋯N1	0.82	1.88	2.594 (2)	145
O3—H3⋯N4	0.82	1.82	2.538 (2)	146
N5—H5⋯N3^i^	0.891 (10)	2.109 (11)	2.991 (3)	171 (2)
N2—H2⋯O3	0.892 (10)	2.097 (10)	2.984 (2)	173 (2)

Symmetry code: (i) 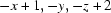.

**Fig. 1.**
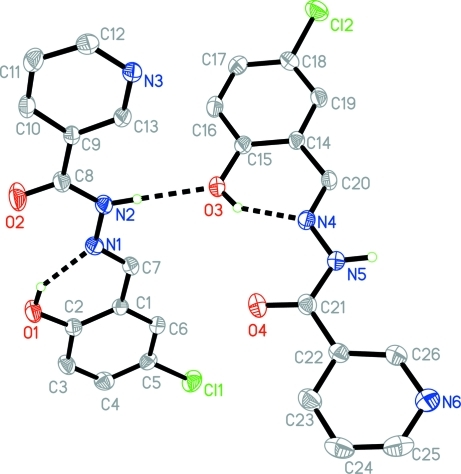
The molecular structure of the compound with 30% probability ellipsoids. The intramolecular O–H···N hydrogen bonds are shown as dashed lines.

**Fig. 2.**
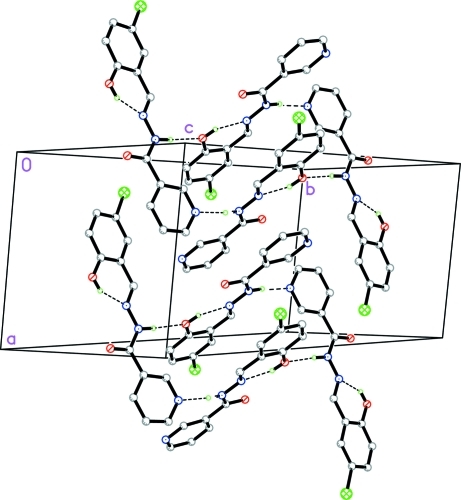
Molecular packing of the compound with hydrogen bonds drawn as dashed lines. Hydrogen atoms not involved in hydrogen bonding have been omitted.

## supplementary crystallographic information

### Comment

The Schiff base compounds show excellent biological properties (Jeewoth *et al.*, 1999; Ren *et al.*, 2002; Eltayeb *et al.*, 2008; Sinha *et al.*, 2008). Moreover, the Schiff base compounds have been widely used as versatile ligands in coordination chemistry (Shivakumar *et al.*, 2008; Prabhakaran *et al.*, 2006; Dhar *et al.*, 2005). We report here the crystal structure of the title compound .In the title compound, Fig. 1, there two independent molecules in the symmetric unit.The dihedral angles between the benzene and pyridine rings are 12.8 (2) and 1.9 (2)°, respectively. All the bond lengths are within normal values (Allen *et al.*, 1987) and comparable to those in other similar compounds (Cui *et al.*, 2007; Jing *et al.*, 2007; Ma *et al.*, 2008; Salhin *et al.*, 2007; Lin *et al.*, 2007; Alhadi *et al.*, 2008; Xue *et al.*, 2008; Wang *et al.*, 2008; Lu, 2008; Diao *et al.*, 2008; Qiu, 2009; Lair *et al.*, 2009*a*,b). The molecules of the title compound are linked into centrosymmetric *R*^4^_4_(26) motifs by N–H···O and N–H···N interactions (Table 1, Fig. 2) (Bernstein *et al.*, 1995).

### Experimental

All the starting materials were obtained with AR grade from Lancaster. 5-Chloro-2-hydroxybenzaldehyde (1.0 mmol, 157.1 mg) and nicotinohydrazide (1.0 mmol, 137.1 mg) were refluxed in a 30 ml methanol solution for 30 min to give a clear yellow solution. Yellow block-shaped single crystals of the compound were obtained by slow evaporation of the solution for five days at room temperature.

### Refinement

H2 and H5 were located from a difference Fourier map and refined isotropically, with the N–H distance restrained to 0.90 (1) Å, and with *U*_iso_ restrained to 0.08 Å^2^. Other H atoms were constrained to ideal geometries, with d(C–H) = 0.93 Å, d(O–H) = 0.82 Å, and with *U*_iso_(H) = 1.2*U*_eq_(C) and 1.5*U*_eq_(O).

### Crystal data

**Table:**

C_13_H_10_ClN_3_O_2_	*F*(000) = 1136
*M**_r_* = 275.69	*D*_x_ = 1.476 Mg m^−^^3^
Monoclinic, *P*2_1_/*n*	Mo *K*α radiation, λ = 0.71073 Å
Hall symbol: -P 2yn	Cell parameters from 2236 reflections
*a* = 9.792 (2) Å	θ = 2.6–24.5°
*b* = 23.358 (3) Å	µ = 0.31 mm^−^^1^
*c* = 10.926 (2) Å	*T* = 298 K
β = 96.848 (2)°	Block, yellow
*V* = 2481.2 (8) Å^3^	0.30 × 0.30 × 0.27 mm
*Z* = 8	

### Data collection

**Table:**

Bruker SMART CCD area-detector diffractometer	5342 independent reflections
Radiation source: fine-focus sealed tube	3193 reflections with *I* > 2σ(*I*)
graphite	*R*_int_ = 0.042
ω scans	θ_max_ = 27.0°, θ_min_ = 1.7°
Absorption correction: multi-scan (*SADABS*; Sheldrick, 1996)	*h* = −12→12
*T*_min_ = 0.913, *T*_max_ = 0.921	*k* = −29→27
14387 measured reflections	*l* = −12→13

### Refinement

**Table:**

Refinement on *F*^2^	Primary atom site location: structure-invariant direct methods
Least-squares matrix: full	Secondary atom site location: difference Fourier map
*R*[*F*^2^ > 2σ(*F*^2^)] = 0.049	Hydrogen site location: inferred from neighbouring sites
*wR*(*F*^2^) = 0.115	H atoms treated by a mixture of independent and constrained refinement
*S* = 1.01	*w* = 1/[σ^2^(*F*_o_^2^) + (0.0466*P*)^2^] where *P* = (*F*_o_^2^ + 2*F*_c_^2^)/3
5342 reflections	(Δ/σ)_max_ < 0.001
351 parameters	Δρ_max_ = 0.22 e Å^−^^3^
2 restraints	Δρ_min_ = −0.24 e Å^−^^3^

### Special details

**Table:**

Geometry. All e.s.d.'s (except the e.s.d. in the dihedral angle between two l.s. planes) are estimated using the full covariance matrix. The cell e.s.d.'s are taken into account individually in the estimation of e.s.d.'s in distances, angles and torsion angles; correlations between e.s.d.'s in cell parameters are only used when they are defined by crystal symmetry. An approximate (isotropic) treatment of cell e.s.d.'s is used for estimating e.s.d.'s involving l.s. planes.
Refinement. Refinement of *F*^2^ against ALL reflections. The weighted *R*-factor *wR* and goodness of fit *S* are based on *F*^2^, conventional *R*-factors *R* are based on *F*, with *F* set to zero for negative *F*^2^. The threshold expression of *F*^2^ > σ(*F*^2^) is used only for calculating *R*-factors(gt) *etc*. and is not relevant to the choice of reflections for refinement. *R*-factors based on *F*^2^ are statistically about twice as large as those based on *F*, and *R*- factors based on ALL data will be even larger.

### Fractional atomic coordinates and isotropic or equivalent isotropic displacement parameters (Å^2^)

**Table:**

	*x*	*y*	*z*	*U*_iso_*/*U*_eq_	
Cl1	−0.31666 (7)	0.15824 (3)	0.49570 (7)	0.0648 (2)	
Cl2	0.67723 (7)	−0.10226 (3)	0.57393 (6)	0.0648 (2)	
N1	0.28860 (19)	0.22587 (8)	0.70899 (16)	0.0414 (5)	
N2	0.4128 (2)	0.20261 (8)	0.75424 (18)	0.0421 (5)	
N3	0.7948 (2)	0.13722 (8)	0.92250 (17)	0.0438 (5)	
N4	0.29718 (18)	0.00154 (8)	0.94498 (16)	0.0387 (4)	
N5	0.2156 (2)	−0.01091 (8)	1.03441 (16)	0.0402 (5)	
N6	−0.0434 (2)	−0.04219 (10)	1.3111 (2)	0.0659 (6)	
O1	0.13564 (17)	0.31528 (6)	0.65672 (16)	0.0520 (4)	
H1	0.2075	0.2981	0.6784	0.078*	
O2	0.50725 (19)	0.29034 (7)	0.76057 (19)	0.0755 (6)	
O3	0.39503 (17)	0.07872 (6)	0.81707 (15)	0.0486 (4)	
H3	0.3484	0.0658	0.8682	0.073*	
O4	0.17570 (18)	0.08396 (7)	1.05029 (15)	0.0598 (5)	
C1	0.0545 (2)	0.21766 (9)	0.63088 (19)	0.0368 (5)	
C2	0.0344 (2)	0.27712 (9)	0.62187 (19)	0.0394 (5)	
C3	−0.0937 (3)	0.29868 (10)	0.5765 (2)	0.0496 (6)	
H3A	−0.1072	0.3381	0.5715	0.059*	
C4	−0.2008 (3)	0.26227 (11)	0.5390 (2)	0.0504 (6)	
H4	−0.2865	0.2770	0.5088	0.060*	
C5	−0.1810 (2)	0.20380 (10)	0.5461 (2)	0.0438 (6)	
C6	−0.0555 (2)	0.18151 (10)	0.59222 (19)	0.0415 (6)	
H6	−0.0439	0.1420	0.5977	0.050*	
C7	0.1862 (2)	0.19285 (10)	0.6792 (2)	0.0411 (6)	
H7	0.1957	0.1534	0.6882	0.049*	
C8	0.5195 (2)	0.23961 (10)	0.7816 (2)	0.0444 (6)	
C9	0.6530 (2)	0.21501 (9)	0.83757 (19)	0.0359 (5)	
C10	0.7653 (3)	0.25086 (10)	0.8548 (2)	0.0483 (6)	
H10	0.7567	0.2890	0.8304	0.058*	
C11	0.8900 (3)	0.23037 (11)	0.9080 (2)	0.0585 (7)	
H11	0.9658	0.2545	0.9224	0.070*	
C12	0.9001 (3)	0.17355 (11)	0.9395 (2)	0.0509 (6)	
H12	0.9849	0.1597	0.9746	0.061*	
C13	0.6739 (2)	0.15881 (9)	0.8743 (2)	0.0425 (6)	
H13	0.5985	0.1343	0.8649	0.051*	
C14	0.4402 (2)	−0.02227 (9)	0.79497 (18)	0.0357 (5)	
C15	0.4588 (2)	0.03497 (9)	0.7643 (2)	0.0380 (5)	
C16	0.5456 (2)	0.04896 (10)	0.6773 (2)	0.0476 (6)	
H16	0.5579	0.0872	0.6572	0.057*	
C17	0.6134 (2)	0.00709 (10)	0.6205 (2)	0.0464 (6)	
H17	0.6724	0.0169	0.5632	0.056*	
C18	0.5936 (2)	−0.04957 (10)	0.6489 (2)	0.0446 (6)	
C19	0.5080 (2)	−0.06441 (9)	0.7344 (2)	0.0414 (6)	
H19	0.4951	−0.1028	0.7523	0.050*	
C20	0.3537 (2)	−0.03840 (9)	0.88893 (19)	0.0395 (5)	
H20	0.3400	−0.0767	0.9076	0.047*	
C21	0.1567 (2)	0.03509 (10)	1.0846 (2)	0.0419 (6)	
C22	0.0660 (2)	0.02299 (9)	1.18211 (19)	0.0401 (5)	
C23	0.0067 (3)	0.06862 (11)	1.2362 (2)	0.0538 (7)	
H23	0.0236	0.1059	1.2124	0.065*	
C24	−0.0774 (3)	0.05834 (13)	1.3256 (2)	0.0651 (8)	
H24	−0.1193	0.0885	1.3624	0.078*	
C25	−0.0986 (3)	0.00294 (14)	1.3595 (2)	0.0657 (8)	
H25	−0.1554	−0.0034	1.4206	0.079*	
C26	0.0382 (3)	−0.03068 (10)	1.2238 (2)	0.0542 (7)	
H26	0.0790	−0.0616	1.1887	0.065*	
H5	0.206 (3)	−0.0477 (5)	1.053 (2)	0.080*	
H2	0.411 (3)	0.1663 (5)	0.779 (2)	0.080*	

### Atomic displacement parameters (Å^2^)

**Table:**

	*U*^11^	*U*^22^	*U*^33^	*U*^12^	*U*^13^	*U*^23^
Cl1	0.0444 (4)	0.0649 (5)	0.0827 (5)	−0.0128 (3)	−0.0025 (4)	0.0037 (4)
Cl2	0.0697 (5)	0.0567 (4)	0.0709 (5)	0.0172 (4)	0.0197 (4)	−0.0037 (3)
N1	0.0369 (12)	0.0387 (11)	0.0479 (11)	0.0041 (9)	0.0020 (9)	0.0047 (9)
N2	0.0351 (12)	0.0342 (11)	0.0552 (12)	0.0012 (9)	−0.0016 (10)	0.0047 (9)
N3	0.0362 (12)	0.0371 (11)	0.0574 (13)	0.0001 (9)	0.0023 (10)	0.0029 (9)
N4	0.0374 (12)	0.0354 (11)	0.0432 (11)	−0.0026 (9)	0.0048 (9)	0.0047 (9)
N5	0.0422 (12)	0.0333 (11)	0.0465 (11)	0.0001 (9)	0.0116 (10)	0.0040 (9)
N6	0.0726 (17)	0.0628 (15)	0.0674 (15)	0.0088 (13)	0.0299 (13)	0.0101 (12)
O1	0.0514 (11)	0.0317 (9)	0.0697 (11)	−0.0002 (8)	−0.0060 (10)	0.0021 (8)
O2	0.0604 (13)	0.0346 (11)	0.1240 (16)	−0.0032 (9)	−0.0194 (12)	0.0196 (10)
O3	0.0549 (11)	0.0292 (9)	0.0649 (11)	−0.0015 (8)	0.0206 (9)	0.0034 (7)
O4	0.0713 (13)	0.0321 (10)	0.0788 (12)	0.0011 (9)	0.0205 (10)	0.0045 (9)
C1	0.0369 (14)	0.0353 (13)	0.0390 (13)	0.0020 (10)	0.0073 (10)	0.0041 (10)
C2	0.0432 (15)	0.0366 (13)	0.0387 (13)	−0.0007 (11)	0.0057 (11)	0.0002 (10)
C3	0.0506 (17)	0.0368 (14)	0.0600 (16)	0.0088 (12)	0.0013 (13)	0.0051 (11)
C4	0.0419 (16)	0.0554 (17)	0.0527 (16)	0.0080 (13)	0.0008 (12)	0.0060 (12)
C5	0.0358 (15)	0.0476 (15)	0.0481 (14)	−0.0051 (11)	0.0056 (11)	0.0018 (11)
C6	0.0430 (15)	0.0354 (13)	0.0467 (14)	−0.0024 (11)	0.0079 (11)	0.0033 (10)
C7	0.0426 (15)	0.0326 (13)	0.0482 (14)	0.0022 (11)	0.0052 (12)	0.0025 (10)
C8	0.0445 (16)	0.0353 (14)	0.0523 (15)	0.0002 (11)	0.0012 (12)	0.0038 (11)
C9	0.0376 (14)	0.0289 (12)	0.0410 (12)	−0.0028 (10)	0.0042 (10)	−0.0003 (9)
C10	0.0498 (17)	0.0308 (13)	0.0635 (16)	−0.0067 (11)	0.0035 (13)	0.0037 (11)
C11	0.0433 (16)	0.0477 (16)	0.0815 (19)	−0.0131 (13)	−0.0043 (14)	0.0003 (14)
C12	0.0367 (15)	0.0520 (17)	0.0625 (16)	0.0016 (12)	0.0000 (12)	0.0000 (12)
C13	0.0338 (14)	0.0358 (13)	0.0571 (15)	−0.0048 (11)	0.0027 (12)	0.0019 (11)
C14	0.0332 (13)	0.0330 (12)	0.0399 (13)	−0.0017 (10)	−0.0006 (10)	0.0050 (10)
C15	0.0335 (13)	0.0355 (13)	0.0444 (13)	−0.0027 (10)	0.0020 (11)	0.0005 (10)
C16	0.0530 (16)	0.0365 (14)	0.0542 (15)	−0.0072 (12)	0.0097 (13)	0.0051 (11)
C17	0.0454 (16)	0.0496 (15)	0.0457 (14)	−0.0044 (12)	0.0121 (12)	0.0040 (12)
C18	0.0449 (15)	0.0427 (14)	0.0455 (14)	0.0065 (11)	0.0029 (12)	0.0007 (11)
C19	0.0445 (15)	0.0330 (13)	0.0459 (13)	0.0020 (11)	0.0025 (11)	0.0062 (10)
C20	0.0417 (14)	0.0281 (12)	0.0482 (14)	−0.0016 (10)	0.0037 (11)	0.0049 (10)
C21	0.0393 (14)	0.0365 (14)	0.0484 (14)	−0.0008 (11)	−0.0004 (11)	−0.0002 (11)
C22	0.0367 (14)	0.0385 (14)	0.0439 (13)	0.0042 (10)	0.0005 (11)	−0.0027 (10)
C23	0.0542 (17)	0.0453 (15)	0.0622 (17)	0.0041 (13)	0.0073 (14)	−0.0108 (12)
C24	0.0600 (19)	0.068 (2)	0.0687 (19)	0.0135 (15)	0.0139 (15)	−0.0213 (15)
C25	0.0514 (18)	0.094 (2)	0.0538 (17)	0.0100 (17)	0.0166 (14)	−0.0020 (16)
C26	0.0606 (18)	0.0439 (16)	0.0619 (16)	0.0083 (13)	0.0230 (14)	−0.0006 (12)

### Geometric parameters (Å, °)

**Table:**

Cl1—C5	1.740 (2)	C7—H7	0.9300
Cl2—C18	1.736 (2)	C8—C9	1.491 (3)
N1—C7	1.276 (3)	C9—C10	1.376 (3)
N1—N2	1.369 (2)	C9—C13	1.381 (3)
N2—C8	1.361 (3)	C10—C11	1.375 (3)
N2—H2	0.892 (10)	C10—H10	0.9300
N3—C12	1.332 (3)	C11—C12	1.372 (3)
N3—C13	1.336 (3)	C11—H11	0.9300
N4—C20	1.278 (3)	C12—H12	0.9300
N4—N5	1.365 (2)	C13—H13	0.9300
N5—C21	1.365 (3)	C14—C15	1.396 (3)
N5—H5	0.891 (10)	C14—C19	1.397 (3)
N6—C25	1.324 (3)	C14—C20	1.456 (3)
N6—C26	1.342 (3)	C15—C16	1.388 (3)
O1—C2	1.354 (3)	C16—C17	1.371 (3)
O1—H1	0.8200	C16—H16	0.9300
O2—C8	1.210 (2)	C17—C18	1.378 (3)
O3—C15	1.361 (2)	C17—H17	0.9300
O3—H3	0.8200	C18—C19	1.373 (3)
O4—C21	1.223 (2)	C19—H19	0.9300
C1—C6	1.394 (3)	C20—H20	0.9300
C1—C2	1.405 (3)	C21—C22	1.493 (3)
C1—C7	1.455 (3)	C22—C26	1.372 (3)
C2—C3	1.387 (3)	C22—C23	1.380 (3)
C3—C4	1.375 (3)	C23—C24	1.372 (3)
C3—H3A	0.9300	C23—H23	0.9300
C4—C5	1.380 (3)	C24—C25	1.369 (4)
C4—H4	0.9300	C24—H24	0.9300
C5—C6	1.374 (3)	C25—H25	0.9300
C6—H6	0.9300	C26—H26	0.9300
			
C7—N1—N2	119.29 (19)	N3—C12—C11	123.3 (2)
C8—N2—N1	116.92 (19)	N3—C12—H12	118.3
C8—N2—H2	125.2 (17)	C11—C12—H12	118.3
N1—N2—H2	116.2 (18)	N3—C13—C9	124.5 (2)
C12—N3—C13	116.7 (2)	N3—C13—H13	117.7
C20—N4—N5	120.75 (18)	C9—C13—H13	117.7
C21—N5—N4	115.52 (18)	C15—C14—C19	118.6 (2)
C21—N5—H5	127.1 (17)	C15—C14—C20	121.4 (2)
N4—N5—H5	117.4 (17)	C19—C14—C20	120.05 (19)
C25—N6—C26	115.5 (2)	O3—C15—C16	117.57 (19)
C2—O1—H1	109.5	O3—C15—C14	122.55 (19)
C15—O3—H3	109.5	C16—C15—C14	119.9 (2)
C6—C1—C2	118.8 (2)	C17—C16—C15	120.7 (2)
C6—C1—C7	119.2 (2)	C17—C16—H16	119.6
C2—C1—C7	122.0 (2)	C15—C16—H16	119.6
O1—C2—C3	117.5 (2)	C16—C17—C18	119.7 (2)
O1—C2—C1	122.7 (2)	C16—C17—H17	120.2
C3—C2—C1	119.8 (2)	C18—C17—H17	120.2
C4—C3—C2	120.5 (2)	C19—C18—C17	120.6 (2)
C4—C3—H3A	119.8	C19—C18—Cl2	120.18 (18)
C2—C3—H3A	119.8	C17—C18—Cl2	119.23 (18)
C3—C4—C5	119.9 (2)	C18—C19—C14	120.5 (2)
C3—C4—H4	120.1	C18—C19—H19	119.7
C5—C4—H4	120.1	C14—C19—H19	119.7
C6—C5—C4	120.6 (2)	N4—C20—C14	118.06 (19)
C6—C5—Cl1	120.01 (19)	N4—C20—H20	121.0
C4—C5—Cl1	119.37 (19)	C14—C20—H20	121.0
C5—C6—C1	120.4 (2)	O4—C21—N5	121.7 (2)
C5—C6—H6	119.8	O4—C21—C22	121.3 (2)
C1—C6—H6	119.8	N5—C21—C22	116.94 (19)
N1—C7—C1	119.2 (2)	C26—C22—C23	116.9 (2)
N1—C7—H7	120.4	C26—C22—C21	124.6 (2)
C1—C7—H7	120.4	C23—C22—C21	118.4 (2)
O2—C8—N2	121.6 (2)	C24—C23—C22	119.3 (2)
O2—C8—C9	121.2 (2)	C24—C23—H23	120.4
N2—C8—C9	117.3 (2)	C22—C23—H23	120.4
C10—C9—C13	116.8 (2)	C25—C24—C23	118.8 (2)
C10—C9—C8	117.9 (2)	C25—C24—H24	120.6
C13—C9—C8	125.3 (2)	C23—C24—H24	120.6
C11—C10—C9	120.0 (2)	N6—C25—C24	124.2 (3)
C11—C10—H10	120.0	N6—C25—H25	117.9
C9—C10—H10	120.0	C24—C25—H25	117.9
C12—C11—C10	118.5 (2)	N6—C26—C22	125.3 (2)
C12—C11—H11	120.8	N6—C26—H26	117.4
C10—C11—H11	120.8	C22—C26—H26	117.4
			
C7—N1—N2—C8	178.3 (2)	C8—C9—C13—N3	178.8 (2)
C20—N4—N5—C21	−177.7 (2)	C19—C14—C15—O3	178.9 (2)
C6—C1—C2—O1	−179.7 (2)	C20—C14—C15—O3	−2.0 (3)
C7—C1—C2—O1	0.3 (3)	C19—C14—C15—C16	−1.3 (3)
C6—C1—C2—C3	0.8 (3)	C20—C14—C15—C16	177.9 (2)
C7—C1—C2—C3	−179.2 (2)	O3—C15—C16—C17	179.9 (2)
O1—C2—C3—C4	179.7 (2)	C14—C15—C16—C17	0.1 (3)
C1—C2—C3—C4	−0.7 (3)	C15—C16—C17—C18	1.0 (4)
C2—C3—C4—C5	−0.2 (4)	C16—C17—C18—C19	−0.8 (4)
C3—C4—C5—C6	1.0 (3)	C16—C17—C18—Cl2	178.62 (18)
C3—C4—C5—Cl1	−178.88 (18)	C17—C18—C19—C14	−0.5 (3)
C4—C5—C6—C1	−1.0 (3)	Cl2—C18—C19—C14	−179.87 (17)
Cl1—C5—C6—C1	178.92 (16)	C15—C14—C19—C18	1.5 (3)
C2—C1—C6—C5	0.1 (3)	C20—C14—C19—C18	−177.7 (2)
C7—C1—C6—C5	−179.9 (2)	N5—N4—C20—C14	−179.66 (18)
N2—N1—C7—C1	−179.88 (18)	C15—C14—C20—N4	−1.5 (3)
C6—C1—C7—N1	176.7 (2)	C19—C14—C20—N4	177.7 (2)
C2—C1—C7—N1	−3.3 (3)	N4—N5—C21—O4	0.5 (3)
N1—N2—C8—O2	−3.9 (3)	N4—N5—C21—C22	179.61 (18)
N1—N2—C8—C9	176.60 (18)	O4—C21—C22—C26	178.9 (2)
O2—C8—C9—C10	−5.6 (3)	N5—C21—C22—C26	−0.2 (3)
N2—C8—C9—C10	173.9 (2)	O4—C21—C22—C23	−2.0 (3)
O2—C8—C9—C13	174.2 (2)	N5—C21—C22—C23	178.9 (2)
N2—C8—C9—C13	−6.3 (3)	C26—C22—C23—C24	−1.1 (4)
C13—C9—C10—C11	−0.9 (3)	C21—C22—C23—C24	179.7 (2)
C8—C9—C10—C11	178.9 (2)	C22—C23—C24—C25	0.8 (4)
C9—C10—C11—C12	2.0 (4)	C26—N6—C25—C24	0.2 (4)
C13—N3—C12—C11	−1.3 (4)	C23—C24—C25—N6	−0.4 (4)
C10—C11—C12—N3	−0.8 (4)	C25—N6—C26—C22	−0.6 (4)
C12—N3—C13—C9	2.5 (3)	C23—C22—C26—N6	1.1 (4)
C10—C9—C13—N3	−1.3 (3)	C21—C22—C26—N6	−179.8 (2)

### Hydrogen-bond geometry (Å, °)

**Table:**

*D*—H···*A*	*D*—H	H···*A*	*D*···*A*	*D*—H···*A*
O1—H1···N1	0.82	1.88	2.594 (2)	145
O3—H3···N4	0.82	1.82	2.538 (2)	146
N5—H5···N3^i^	0.89 (1)	2.11 (1)	2.991 (3)	171 (2)
N2—H2···O3	0.89 (1)	2.10 (1)	2.984 (2)	173 (2)

Symmetry codes: (i) −*x*+1, −*y*, −*z*+2.
